# Biochemical, Clinical, and Genetic Characteristics of Short/Branched Chain Acyl-CoA Dehydrogenase Deficiency in Chinese Patients by Newborn Screening

**DOI:** 10.3389/fgene.2019.00802

**Published:** 2019-08-28

**Authors:** Yiming Lin, Hongzhi Gao, Chunmei Lin, Yanru Chen, Shuang Zhou, Weihua Lin, Zhenzhu Zheng, Xiaoqing Li, Min Li, Qingliu Fu

**Affiliations:** ^1^Neonatal Disease Screening Center, Quanzhou Maternal and Children’s Hospital, Quanzhou, China; ^2^Department of Central Laboratory, 2nd Affiliated Hospital of Fujian Medical University, Quanzhou, China; ^3^Department of Neonatal Intensive Care Unit, Quanzhou Maternal and Children’s Hospital Quanzhou, China; ^4^Zhejiang Biosan Biochemical Technologies Co., Ltd, Hangzhou, China

**Keywords:** short/branched chain acyl-CoA dehydrogenase deficiency, 2-methylbutyryl-CoA dehydrogenase deficiency, *ACADSB* gene, newborn screening, isoleucine catabolism

## Abstract

Short/branched chain acyl-CoA dehydrogenase deficiency (SBCADD) is an autosomal recessive disorder of impaired isoleucine catabolism caused by mutations in the *ACADSB* gene. There are limited SBCADD cases worldwide and to date no Chinese patients with SBCADD have been reported. The aim of this study was to investigate the biochemical, clinical information, and genotypes of twelve patients with SBCADD in China for the first time. The estimated incidence of SBCADD was 1 in 30,379 in Quanzhou, China. The initial newborn screening (NBS) results revealed that all patients showed slightly or moderately elevated C5 concentrations with C5/C2 and C5/C3 ratios in the reference range, which has the highest risk of being missed. All patients who underwent urinary organic acid analysis showed elevation of 2-methylburtyrylglycine in urine. All patients were asymptomatic at diagnosis, and had normal growth and development during follow-up. Eight different variants in the *ACADSB* gene, including five previously unreported variants were identified, namely c.596A > G (p.Tyr199Cys), c.653T > C (p.Leu218Pro), c.746del (p.Pro249Leufs*15), c.886G > T (p.Gly296*) and c.923G > A (p.Cys308Tyr). The most common variant was c.1165A > G (33.3%), followed by c.275C > G (20.8%). All previously unreported variants may cause structural damage and dysfunction of SBCAD, as predicted by bioinformatics analysis. Thus, our findings indicate that SBCADD may be more frequent in the Chinese population than previously thought and newborn screening, combined with genetic testing is important for timely diagnosis. Although the clinical course of Chinese patients with SBCADD is likely benign, longitudinal follow-up may be helpful to better understand the natural history of SBCADD.

## Introduction

Short/branched chain acyl-CoA dehydrogenase deficiency (SBCADD, OMIM # 610006), also known as 2-methylbutyryl-CoA dehydrogenase deficiency (2-MBCDD), is an autosomal recessive disorder of impaired isoleucine catabolism (Andresen et al., 2000; Gibson et al., 2000). The causative gene, *ACADSB* (MIM 600301), was mapped to chromosome 10q26.13, comprises 11 exons and encodes a SBCAD precursor protein of 432 amino acids (Arden et al., 1995). The clinical manifestations of SBCADD can be variable, including developmental delay, intellectual disability, seizures, muscular atrophy, hypotonia, autism and neonatal crisis (Akaboshi et al., 2001; Korman et al., 2001; Korman, 2006; Kanavin et al., 2007). However, patients with SBCADD ascertained *via* expanded NBS were reported to have normal development and no obvious health problems, especially in the Hmong population (Matern et al., 2003; Alfardan et al., 2010). Currently, therapeutic strategies for patients with SBCADD are poorly defined because there are no conclusive data. However, careful instructions regarding risks for metabolic stress and fasting avoidance, along with clinical monitoring are warranted (Knerr et al., 2012).

Most patients with SBCADD showed elevated concentrations of 2-methylbutyrylcarnitine (C5), which are detectable at NBS by tandem mass spectrometry (MS/MS). Nevertheless, MS/MS-based NBS may lack sensitivity as some patients with C5-acylcarnitine concentrations within the reference range cannot be detected through NBS. Furthermore, the C5 value was also elevated in isovaleric acidemia (IVA, OMIM # 243500) as isovalerylcarnitine and 2-methylbutyrylcarnitine presenting the same mass to charge are indistinguishable by MS/MS. Thus, urinary organic acid analysis and genetic testing are necessary for differential diagnosis (Madsen et al., 2006; Sass et al., 2008).

SBCADD is highly prevalent in the Hmong ethnic population, whereas only a small number of non-Hmong patients have been reported (van Calcar et al., 2007; Van Calcar et al., 2013). The majority of China’s population is dominated with the Han nationality, however, no patients with SBCADD have been reported so far. Herein, we present for the first time the biochemical, clinical information, and genotypes of twelve patients with SBCADD from mainland of China, which may contribute to current research.

## Materials and Methods

### Patients and Auxiliary Analysis

A total of 364,545 newborns (209,136 males and 155,409 females) were screened by MS/MS at Quanzhou Maternity and Children’s Hospital between January 2014 and November 2018. Blood samples for NBS were collected between 72 h and 7 days after birth. The concentrations of C5-acylcarnitine, C5/acetylcarnitine (C2) and C5/propionylcarnitine (C3) on dried blood spot (DBS) filter paper cards were determined by using ACQUITY TQD (Waters, Milford, MA, USA) and a NeoBase non-derivatized MS/MS kit (PekinElmer, USA). Among the 364,545 newborns, 351 showed elevated C5-acylcarnitine concentrations in NBS, 44 newborns were involved with confirmatory testing and had positive results in the second test. Otherwise, twelve patients genetically diagnosed with SBCADD were followed up in our hospital. Nine patients’ urine samples were then collected for urine organic acid analysis by gas chromatography-mass spectrometry (7890B/5977A, Agilent Technologies, Santa Clara, CA, USA). In addition, assessment of growth, development, and clinical manifestation was conducted, and the patients were followed and evaluated every 3–6 months. This study was approved by the Ethical Committee of Quanzhou Maternity and Children’s Hospital. Written informed consent was obtained from all parents of the patients and control subjects, who agreed to participate in this study with the intent of using their medical data for scientific research and publication.

### Next-Generation Sequencing (NGS) and Variants Analysis

DBS or peripheral whole blood of twelve patients and their parents, as well as 100 control subjects, were referred to the laboratory. Genomic DNA was extracted using Qiagen Blood DNA mini kits (Qiagen, Hilden, Germany) according to the manufacturer’s protocol. DNA samples of the probands were quantified using a Qubit^®^ dsDNA HS Assay Kit (Invitrogen, Carlsbad, CA, USA) and then were sequenced using NGS. The target sequencing panel of 94 known genes involved in inherited metabolic disorders including abnormal C5-carnitine related genes (*ACADSB* and *IVD*) was used. Genomic DNA was sheared to an approximate mean fragment length of 200-base pair (bp) using the Covaris LE220 ultrasonicator (Covaris, Woburn, MA). Sheared DNA was used for library preparation of targeted regions by multiplex polymerase chain reaction (PCR). The library concentration and amplicon size were determined using an Agilent High Sensitivity DNA Kit (Agilent, Santa Clara, CA, USA). The prepared sample libraries were sequenced using an Illumina NextSeq 500 platform (Illumina Inc., San Diego, CA, USA) in paired end mode, generating 150-bp paired end reads. The data were analyzed using NextSeq 500 Reporter and raw sequencing data were processed for subsequent bioinformatics analysis. The workflows of variant filtering and bioinformatics analysis were as previously described (Lin et al., 2018).

To explore the structural changes in the protein, homology modeling was used to build the three-dimensional (3D) models of ACADSB using Swiss Model Workspace with the PDB accession number 2JIF. The PDB files were submitted to the Swiss-pdb Viewer 4.0 to view the 3D-structure. The assessment of pathogenicity of these previously unreported variants was performed based on the American College of Medical Genetics and Genomics (ACMG) guidelines (Richards et al., 2015). Additionally, one hundred healthy newborns from our center were randomly selected to assess the variant frequencies in normal controls. All control subjects with expanded NBS had results in the reference range.

### Sanger Sequencing

The variants identified by NGS were further validated by Sanger sequencing of the patients and their parents. The exonic and flanking intronic sequences of the *ACADSB* gene were amplified by PCR. PCR products were sequenced directly on an ABI Prism 3500 automatic sequencer (Applied Biosystems, Foster City, CA, USA). All primers are listed in **Supplementary File 1: Table S1**.

### Statistical Analysis

Statistical analysis was performed using SPSS 11.0 version (SPSS Inc., Chicago, IL, USA). A confidence interval of 95% was selected.

## Results

### Biochemical and Clinical Characteristics

Twelve confirmed SBCADD cases (seven females and five males) were from twelve unrelated families, most from the Han ethic population. The initial NBS results revealed that all patients showed slightly or moderately elevated C5-carnitine concentrations (C5 cutoff value: 0.03–0.35 μmol/L), along with C5/C2 and C5/C3 ratios in the reference range (cutoff value: C5/C2: 0–0.04, C5/C3: 0.02–0.42). During follow-up, the patients’ C5-carnitine concentrations presented variable levels but were always higher than the cut-off values, with the sole exception of one patient (no. 11) falling into the normal range at 31 days. Most of the C5/C2 and C5/C3 ratios were higher than the cut-off values. Increased concentrations of urinary 2-methylburtyrylglycine (2-MBG), in the absence of isovalerylglycine excretion were observed in all nine patients who underwent urinary organic acid analysis. All twelve patients in our study had normal growth and development and remained asymptomatic during follow-up (between ages 3–20 months). The biochemical, clinical features, and genotypes of all twelve patients are summarized in **Table 1**.

**Table 1:** Biochemical phenotype and genotype of twelve newborns with SBCADD.

Patient no.	Sex	Age^a^	Ethnic	NBS			Follow-up			Urine 2-MBG (mmol/mol creatinine)^e^	Genotype^f^	
				C5(μmol/L)^b^	C5/C2^c^	C5/C3^d^	C5(μmol/L)	C5/C2	C5/C3			
1	F	1 y, 8 m	Han	0.38	0.03	0.38	0.56/0.83 (20 d/34 d)	0.07/0.09 (20 d/34 d)	1.04/0.87 (20 d/34 d)	24.84	c.1165A > G c.1165A > G	p.Met389Val p.Met389Val
2	F	1 y, 6 m	Han	0.67	0.03	0.16	0.74 (15 d)	0.06 (15 d)	0.47(15 d)	31.73	c.655G > A **c.923G > A**	p.Val219Met **p.Cys308Tyr**
3	F	1 y, 3 m	Han	0.51	0.02	0.28	0.79/0.44 (19 d/7 m)	0.05/0.02 (19 d/7 m)	0.87/0.47 (19 d/7 m)	ND	**c.923G > A** **c.923G > A**	**p.Cys308Tyr** **p.Cys308Tyr**
4	F	1 y, 2 m	Hmong	0.69	0.04	0.26	1.66/0.86 (13 d/21 d)	0.17/0.09 (13 d/21 d)	0.97/0.66 (13 d/21 d)	40.55	c.1165A > G c.1165A > G	p.Met389Val p.Met389Val
5	M	1 y, 2 m	Han	0.37	0.02	0.23	0.88/1.01 (15 d/26 d)	0.09/0.08 (15 d/26 d)	0.54/0.5 (15 d/26 d)	15.19	c.275C > G **c.653T > C**	p.Ser92* **p.Leu218Pro**
6	F	1 y, 1 m	Han	0.38	0.03	0.39	0.53/0.5 (19 d/31 d)	0.05/0.07 (19 d/31 d)	0.48/0.56 (19 d/31 d)	79.49	**c.746del** **c.746del**	p.Pro249Leufs*15 p.Pro249Leufs*15
7	F	10 m	Han	0.64	0.04	0.26	0.6/0.49 (17 d/30 d)	0.1/0.09 (17 d/30 d)	1.36/1.11 (17 d/30 d)	68.73	c.655G > A **c.886G > T**	p.Val219Metp.Gly296*
8	M	8 m	Han	0.38	0.02	0.18	0.43 (25 d)	0.05(25 d)	0.3(25 d)	ND	c.655G > A **c.596A > G**	p.Val219Met **p.Tyr199Cys**
9	M	6 m	Han	0.44	0.02	0.28	0.64 (14 d)	0.09 (14 d)	1.08 (14 d)	ND	c.275C > G c.275C > G	p.Ser92* p.Ser92*
10	F	5 m	Han	0.41	0.03	0.35	0.55/0.58 (13 d/25 d)	0.06/0.08 (17 d/25 d)	0.71/0.92 (17 d/25 d)	39.69	c.275C > G c.275C > G	p.Ser92* p.Ser92*
11	M	5 m	Han	0.37	0.04	0.21	0.39/0.31 (14 d/31 d)	0.07/0.06 (17 d/31 d)	0.42/0.34 (14 d/31 d)	21.26	c.1165A > G c.1165A > G	p.Met389Val p.Met389Valw
12	M	3 m	Bouyei	0.36		0.17	0.51/0.38 (15 d/36 d)	0.08/0.05 (15 d/36 d)	0.76/0.43 (15 d/36 d)	18.47	c.1165A > G c.1165A > G	p.Met389Val p.Met389Val

^a^As of December 2018, d: day, m: month, y: year.

^b^Reference range: 0.03–0.35 μmol/L; ^c^reference range: 0–0.04; ^d^reference range: 0.02–0.42; ^e^2-MBG: 2-methylburtyrylglycine, reference range: < 0.2 mmol/mol creatinine.

^f^The previously unreported variants of this study are in boldface type.

ND, not determined.

### Genetic Studies and Variant Analysis

**Figure 1:**
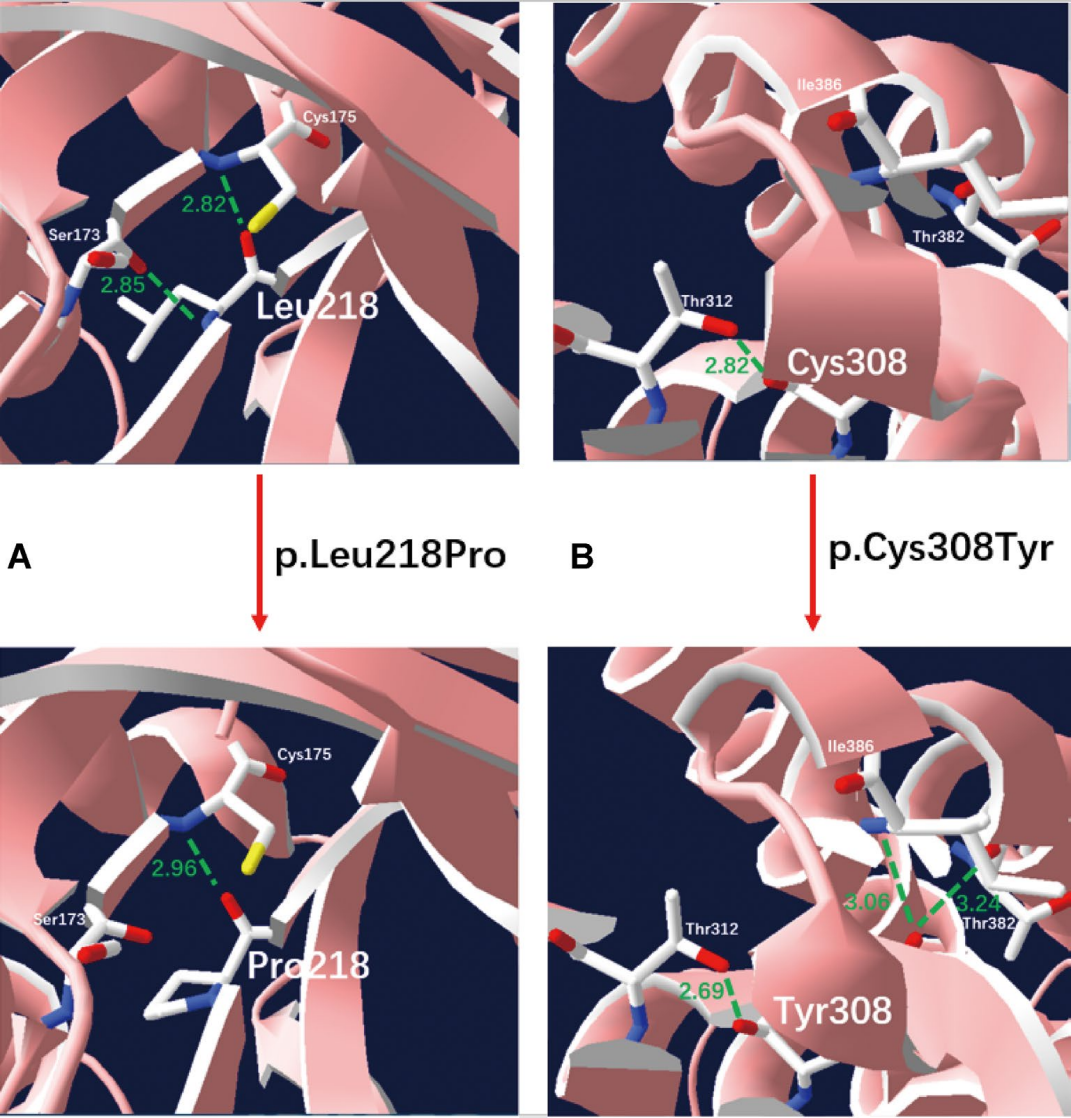
Three-dimensional structure analysis modeling of wild-type and SBCAD mutant products. Green dashed lines represent hydrogen bonds and the green number shows the hydrogen bond distances. **(A)** The p.Leu218Pro variant may affect the *ACADSB* quaternary structure by losing intramolecular hydrogen bonding with 173-Ser. Thus, the protein product would be unstable and most likely be rapidly degraded. **(B)** The p.Cys308Tyr variant may alter the side chain conformations of the residues in the FAD cofactor binding cavity by inducing intramolecular hydrogen bonding with 382-Thr and 386-Ile, which may block the binding of FAD.

We applied our target sequencing panel, which included the abnormal C5-carnitine related genes (*ACADSB* and *IVD*) to all twelve patients with SBCADD and achieved high-quality results. On average, the mean coverage of the target regions was 530.14X. Coverage of targeted regions for >10X reads ranged from 99.75% to 99.91% and >20X reads from 99.50% to 99.91%. In total, eight different variants in the *ACADSB* gene were identified from 24 mutant alleles of twelve patients with SBCADD (**Supplementary File 2: Figure S1**). These patients have two allelic variants, eight of which were homozygotes and four were heterozygotes. Three variants have been reported previously, namely c.275C > G (p.Ser92*), c.655G > A (p.Val219Met), and c.1165A > G (p.Met389Val), the remaining five variants were previously unreported, including three missense variants c.596A > G (p.Tyr199Cys), c.653T > C (p.Leu218Pro), c.923G > A (p.Cys308Tyr), one nonsense variant c.886G > T (p.Gly296*) and one deletion variant c.746del (p.Pro249Leufs*15). The most frequently variant was c.1165A > G (p.Met389Val), with an allelic frequency of 8/24 (33.3%), followed by c.275C > G (p.Ser92*) 5/24 (20.8%). These previously unreported variants have not been recorded in disease databases such as HGMD, ClinVar, and LOVD, and were not detected in 100 control subjects. All previously unreported missense variants were predicted to be deleterious *in silico* by SIFT, Polyphen-2, PROVEAN, and the Mutation Taster software (**Table 2**). Additionally, sequence alignments of different species revealed that the amino acid residues at positions 199, 218, and 308 in the SBCAD protein are strictly conserved (**Supplementary File 3: Figure S2**). Furthermore, although 3D-modeling analysis showed that the p.Tyr199Cys variant does not cause any significant change of the intramolecular hydrogen bonding (the corresponding 3D-modeling results are therefore not shown), since this variant substitutes a hydrophobic Tyrosine for a hydrophilic Cysteine, this might result in abnormal folding of ACADSB. The p.Leu218Pro variant may affect the ACADSB quaternary structure by losing intramolecular hydrogen bonding with 173-Ser. Thus, the protein product would be unstable and most likely be rapidly degraded (**Figure 1A**). The p.Cys308Tyr variant may alter the side chain conformations of the residues in the FAD cofactor binding cavity by inducing intramolecular hydrogen bonding with 382-Thr and 386-Ile, which may block the binding of FAD (**Figure 1B**). No further studies were performed on p.Gly296* and p.P249Lfs*15 since both variants would lead to truncated proteins that would potentially affect protein function. According to ACMG recommendations, three variants c.596A > G (p.Tyr199Cys), c.653T > C (p.Leu218Pro), and c.923G > A (p.Cys308Tyr) were classified as variants of uncertain significance (VUS), the c.886G > T (p.Gly296*) and c.746del (p.Pro249Leufs*15) were classified as likely pathogenic (LP) (**Supplementary File 4: Table S2**).

**Table 2:** *In silico* prediction and analysis of the previously unreported *ACADSB* gene variants.

No	Location	Nucleotide change	Protein change	SIFT^a^	PolyPhen-2^b^	PROVEAN^c^	Mutation Taster^d^	HGMD^e^	ClinVar^f^	LOVD^g^	dbSNP^h^	Freq in ExAC^i^	Freq in 1000 Genome^j^
1	Exon 5	c.596A > G	p.Tyr199Cys	0	0.996	-7.57	0.999	ND	ND	ND	ND	ND	ND
2	Exon 5	c.653T > C	p.Leu218Pro	0	0.999	-6.04	0.999	ND	ND	ND	ND	ND	ND
3	Exon 6	c.746del	p.Pro249Leufs*15	N/A	N/A	N/A	1	ND	ND	ND	ND	8.24E-05	ND
4	Exon 7	c.886G > T	p.Gly296*	N/A	N/A	N/A	1	ND	ND	ND	ND	ND	ND
5	Exon 8	c.923G > A	p.Cys308Tyr	0	0.997	-7.87	0.999	ND	ND	ND	rs770456976	2.48E-05	ND

The reference sequence used in this study was based on the NCBI37/hg19 assembly of the human genome. NM_001609 was employed as reference sequence for ACADSB.

ND, no data.

N/A,t not available.

^a^SIFT: http://sift.jcvi.org/, ^b^PolyPhen-2: http://genetics.bwh.harvard.edu/pph2/, ^c^PROVEAN: http://provean.jcvi.org/index.php, ^d^MutationTaster: http://www.mutationtaster.org/, ^e^HGMD: http://www.hgmd.cf.ac.uk/ac/index.php, ^f^ClinVar: https://www.ncbi.nlm.nih.gov/clinvar/, ^g^Leiden Open Variation Database http://www.lovd.nl/3.0/home, ^h^dbSNP: https://www.ncbi.nlm.nih.gov/projects/SNP/, ^i^ExAC: http://exac.broadinstitue.org/, ^j^1000 Genome Project: http://www.1000genomes.org/.

## Discussion

This study reported twelve patients with SBCADD in the Chinese population for the first time. The estimated incidence of SBCADD in the Chinese population was 1/30,379 (95% CI: 1/69,964-1/19,402) at a single screening center. Our findings indicate that SBCADD may be more frequent in the Chinese population than previously thought. It is notable that some patients with C5-carnitine concentrations below the current cutoff may go undetected, as previously described (Van Calcar et al., 2013). Hence the true incidence of SBCADD in our study is probably underestimated. In comparison, Van Calcar,SC *et al*. reported that the frequency of SBCADD in Hmong–American was up to 1:132, while the prevalence in non-Hmong population in Wisconsin was 1:540,780 (Van Calcar et al., 2013). Therefore, the prevalence of SBCADD shows regional and ethnic differences. Clearly, lowering the C5 cut-off value will increase the detection rate of SBCADD, but it will also increase the false positive rate. Since C5 is also the hallmark of IVA, a disorder of leucine catabolism, setting the C5 cut-off value is challenging.

All patients were identified *via* NBS with elevated concentrations of C5-carnitine, where the initial C5-carnitine concentrations ranged from an increase of 1–2 fold. In contrast, the C5 concentrations of patients with IVA increased more significantly, although the C5 values of IVA and SBCADD may overlap (Region 4 Genetics Collaborative MS/MS Project, 2012). It is worth noting that all the initial C5/C2 and C5/C3 ratios in our samples were within the reference range. This is in agreement with the previous notion that the absence of elevated C5 ratios on NBS are highly suggestive of SBCADD (Van Calcar et al., 2013). Thus our research shows once again that combining initial C5 concentrations with C5 ratios helps distinguish between these two disorders. All patients who underwent urinary organic acid analysis showed elevation of 2-MBG in the urine, indicating that 2-MBG may be a sensitive indicator, despite the limited number of patients presented.

Based on a recent literature review (Porta et al., 2019), SBCADD is symptomatic only in about 10% of reported patients. Notably, these patients’ clinical manifestations cannot be attributed solely to SBCADD because there may be confounding factors such as the presence of other diseases or metabolic disorders that have not yet been diagnosed. Therefore, the small number of patients that have clinical symptoms appear to be coincidental due to the association between SBCADD and the presence of symptoms has not yet been established. In addition, most current evidence supports the idea that SBCADD is a biochemical phenotype rather than a disease, although a few reports claim that it is unsafe to consider SBCADD as a “non-disease” (Alfardan et al., 2010; Knebel et al., 2012; Van Calcar et al., 2013; Nochi et al., 2017).

The inclusion of SBCADD in NBS programs remains controversial. Nevertheless, it is noteworthy that SBCADD is extremely important for differential diagnosis of IVA, a metabolic disorder that can cause significant morbidity and mortality. Therefore, it is imprudent to completely disregard SBCADD from NBS programs. Given this, SBCADD is currently included in the recommended uniform screening panel (RUSP) as a secondary disorder. In contrast, SBCADD was not included in NBS programs of many countries when they considered cost-effectiveness and clinical utility, resulting in many patients with SBCADD not being diagnosed and less likely to be followed during development. Currently, we have retained SBCADD in our NBS programs. Similar to most SBCADD cases described so far, all patients in this study had normal growth and development and remained asymptomatic during follow-up. However, the clinical follow-up time was relatively short, we therefore cannot rule out the possibility of clinical manifestations in later life. Because of insufficient research and that the clinical course of SBCADD in the general population is not very predictable, longitudinal follow-up may be helpful to further define the natural history of SBCADD.

Until now, only fifteen variants in the *ACADSB* gene have previously been described in the literature to cause SBCADD (Andresen et al., 2000; Gibson et al., 2000; Matern et al., 2003; Korman et al., 2005; Madsen et al., 2006; Sass et al., 2008; Alfardan et al., 2010; Lanthaler et al., 2014; Deciphering Developmental Disorders Study, 2015; Porta et al., 2019). In the present study, eight different variants, including five previously unreported variants in the *ACADSB* gene were identified. The most common variant was c.1165A > G (33.3%), followed by c.275C > G (20.8%). The c.1165A > G variant was reported previously as a founder mutation in the Hmong ethnic group that was originally from southwestern China; the Hmong population ranks fourth among Chinese ethnic minorities. In this study, the c.1165A > G homozygous variant was found in two Han patients, one Bouyei patient and one Hmong patient, which suggest a possible founder effect in the Chinese population. The c.275C > G and c.655G > A variants were reported previously in an asymptomatic Taiwan infant identified by NBS (Liu et al., 2007). For previously unreported variants, the main evidence for the classification of variants are as follows: (i) all variants were not present or have an extremely low allelic frequency in dbSNP, ExAC, and 1000 Genome, and are not listed in the disease databases such as HGMD, ClinVar, and LOVD; (ii) in all variants, a highly conserved amino acid of SBCAD was changed, and was predicted to have a damaging effect on the SBCAD protein using different bioinformatics analysis programs; and (iii) 3D-modeling analysis showed that these missense variants may affect the ACADSB quaternary structure and result in the production of an unstable ACADSB protein or block the binding of the FAD cofactor, which may affect enzyme activity. The limitation of our study is that further definite proof such as functional assays were not performed because of limited resources, although all three previously unreported missense variants were predicted as probably disease-causing by bioinformatics analysis and the other two previously unreported variants would lead to truncated proteins and potentially affect protein function.

In conclusion, this is the first report of biochemical, clinical, and genetic analysis of SBCADD in the Chinese population. Our findings indicate a high incidence of SBCADD among the Chinese population. Five previously unreported variants in the *ACADSB* gene were identified, which expanded the *ACADSB* variation spectrum. Although the clinical course of our patients are likely benign, longitudinal follow-up may be helpful to better understand the natural history of SBCADD.

## Author Contributions

YL designed the study, performed experimental work, paper writing and drafting; ML carried out the genetic tests, mutation analysis and paper editing; HG, SZ and XL participated in manuscript preparation; CL, YC, WL and ZZ followed the patients and collected the clinical data; QF was the mentor who designed and guided the research study. All authors read and approved the final manuscript.

## Funding

This study was supported by the Quanzhou Municipal Science and Technology Plan Project (Grant No. 2018N085S).

## Conflict of Interest Statement

Author ML was employed by Zhejiang Biosan Biochemical Technologies Co., Ltd, China. The remaining authors declare that the research was conducted in the absence of any commercial or financial relationships that could be construed as a potential conflict of interest.

## Abbreviations

SBCADD, Short/branched chain acyl-CoA dehydrogenase deficiency; 2-MBCDD, 2-methylbutyryl-CoA dehydrogenase deficiency; NBS, Newborn screening; MS/MS, Tandem mass spectrometry; IVA, Isovaleric acidemia; DBS, Dried blood spots; NGS, Next-generation sequencing; PCR, Polymerase chain reaction; 3D, Three-dimensional; ACMG, American College of Medical Genetics Association of Clinical Genetics; 2-MBG, 2-methylburtyrylglycine; VUS, Variants of uncertain significance; LP, Likely pathogenic.

## Supplementary Material

Supplementary file 1: Table S1 List of primers used for Sanger sequencing.

Supplementary file 2: Figure S1 Validation of *ACADSB* variants by Sanger sequencing (the variant is indicated with a red arrow).

Supplementary file 3: Figure S2 Multiple sequence alignment using ClustalX. The amino acid residues at positions 199, 218, and 308 in the SBCAD protein (highlighted in box) are strictly conserved among various species.

Supplementary file 4: Table S2 Evidence and classification of previously unreported variants.
