# Supplementary material for: Biochemical, Clinical, and Genetic Characteristics of Short/Branched Chain Acyl-CoA Dehydrogenase Deficiency in Chinese Patients by Newborn Screening
Source: Front Genet. 2019 Aug 28;10:802. doi: 10.3389/fgene.2019.00802 (PMC6727870; doi:10.3389/fgene.2019.00802)
Supplement: Supplementary file 1: Table S1 — List of primers used for Sanger sequencing. [file Table_1.doc]

**Table S1：List of the primers used for Sanger sequencing**

| Variants | Exon/Intron | Primer-Forward | Primer-Reverse |
| --- | --- | --- | --- |
| c.275C>G | Exon 3 | TGTAAAACGACGGCCAGTTTTGTGCTTTCTTTATTG | CAGGAAACAGCTATGACCTTATGCTATCATTACAGTCC |
| c.596A>G | Exon 5 | TGTAAAACGACGGCCAGTCCTGGGAGACGGAGCAAG | CAGGAAACAGCTATGACCGGAAACAGAAGGAGGAAAAGATA |
| c.653T>C | Exon 5 | TGTAAAACGACGGCCAGTCCTGGGAGACGGAGCAAG | CAGGAAACAGCTATGACCGGAAACAGAAGGAGGAAAAGATA |
| c.655G>A | Exon 5 | TGTAAAACGACGGCCAGTCCTGGGAGACGGAGCAAG | CAGGAAACAGCTATGACCGGAAACAGAAGGAGGAAAAGATA |
| c.746del | Exon 6 | TGTAAAACGACGGCCAGTCTTTTTACTTAAACTCTTCA | CAGGAAACAGCTATGACCGATTAGTCTACGATACCCA |
| c.886G>T | Exon 7 | TGTAAAACGACGGCCAGTGTAGAGGGAGACACAGATG | CAGGAAACAGCTATGACCTTGAAAGAAATAATAAGCC |
| c.923G>A | Exon 8 | TGTAAAACGACGGCCAGTTAACACACACCTCACCAC | CAGGAAACAGCTATGACCTATTCTTTCCTCCCCTAC |
| c.1165A>G | Exon 10 | TGTAAAACGACGGCCAGTAAATGGTATGGAGAATGG | CAGGAAACAGCTATGACCTTTGAAGGACGAGGTAAG |
