# Supplementary figures and images for: Biochemical, Clinical, and Genetic Characteristics of Short/Branched Chain Acyl-CoA Dehydrogenase Deficiency in Chinese Patients by Newborn Screening
Source: Front Genet. 2019 Aug 28;10:802. doi: 10.3389/fgene.2019.00802 (PMC6727870; doi:10.3389/fgene.2019.00802)

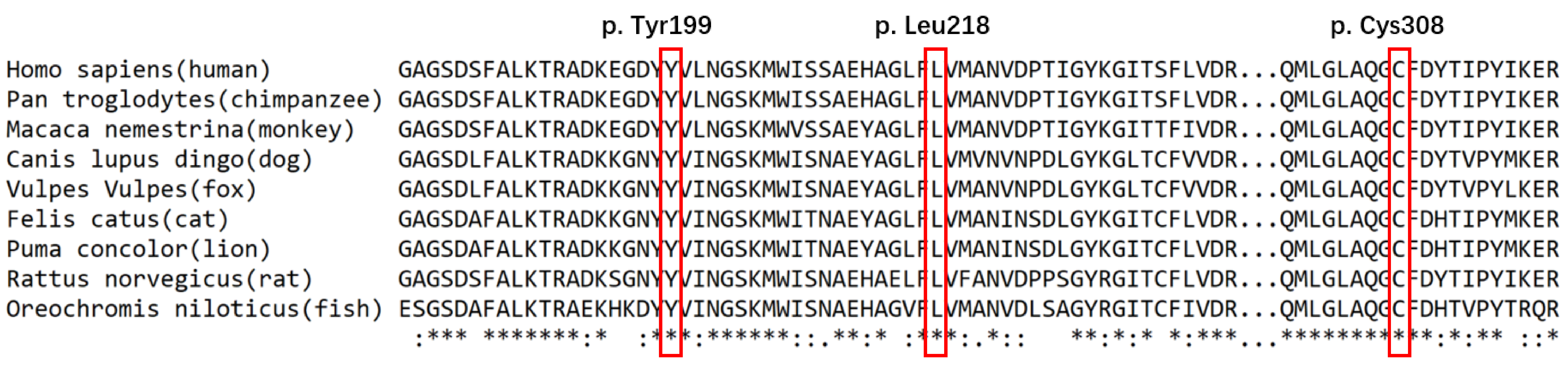

Supplement: Supplementary file 3: Figure S2 — Multiple sequence alignment using ClustalX. The amino acid residues at positions 199, 218, and 308 in the SBCAD protein (highlighted in box) are strictly conserved among various species. [file Image_2.tif]
