# Supplementary material for: Biochemical, Clinical, and Genetic Characteristics of Short/Branched Chain Acyl-CoA Dehydrogenase Deficiency in Chinese Patients by Newborn Screening
Source: Front Genet. 2019 Aug 28;10:802. doi: 10.3389/fgene.2019.00802 (PMC6727870; doi:10.3389/fgene.2019.00802)
Supplement: Supplementary file 4: Table S2 — Evidence and classification of previously unreported variants. [file Table_2.doc]

**Table S2:** Evidence and Classification of previously unreported variants

| No | Nucleotide  change | Protein  change | PVS1^a^ | PM2^b^ | | PP3^c^ | Combining evidence | Classification |
| --- | --- | --- | --- | --- | --- | --- | --- | --- |
|  |  |  |  | Freq in ExAC | Freq in 1000 Genome |  |  |  |
| 1 | c.596A>G | p.Tyr199Cys | N/A | ND | ND | Damaging | PM2+PP3 | VUS |
| 2 | c.653T>C | p.Leu218Pro | N/A | ND | ND | Damaging | PM2+PP3 | VUS |
| 3 | c.746del | p.Pro249Leufs*15 | Truncating variant | 8.24E-05 | ND | N/A | PVS1+PM2 | LP |
| 4 | c.886G>T | p.Gly296* | Truncating variant | ND | ND | N/A | PVS1+PM2 | LP |
| 5 | c.923G>A | p.Cys308Tyr | N/A | 2.48E-05 | ND | Damaging | PM2+PP3 | VUS |

Evidence and Classification according to the American College of Medical Genetics and Genomics (ACMG) guidelines.

^a^PVS1: Predicted null variant in a gene where LOF is a known mechanism of disease.

^b^PM2: Absent from controls (or at extremely low frequency if recessive) in population databases such as ExAC and 1000 Genome.

^c^PP3: Multiple lines of computational evidence support a deleterious effect on the gene /gene product.

ND: no data.

N/A: not available.

VUS: Variants of uncertain significance.

LP: Likely pathogenic.
