# Supplementary material for: Biochemical, Clinical, and Genetic Characteristics of Short/Branched Chain Acyl-CoA Dehydrogenase Deficiency in Chinese Patients by Newborn Screening
Source: Front Genet. 2019 Aug 28;10:802. doi: 10.3389/fgene.2019.00802 (PMC6727870; doi:10.3389/fgene.2019.00802)

Confidence Intervals for One Proportion - New

Numeric Results for One-Sided Upper-Limit Confidence Intervals for One Proportion
Confidence Interval Formula: Exact (Clopper-Pearson)

Confidence Level	Sample Size (N)	Target Distance from P to Upper Limit	Actual Distance from P to Upper Limit	Proportion (P)	Lower Limit	Upper Limit	Distance from P to Limit if P = 0.5
0.950	169595	0.002	0.002	0.500	0	0.502	0.002
0.990		0.002	0.002	0.500	0	0.502	

References

Fleiss, J. L., Levin, B., Paik, M.C. 2003. Statistical Methods for Rates and Proportions. Third Edition. John Wiley & Sons. New York.

Newcombe, R. G. 1998. 'Two-Sided Confidence Intervals for the Single Proportion: Comparison of Seven Methods.' Statistics in Medicine, 17, pp. 857-872.

Report Definitions

Confidence level is the proportion of confidence intervals (constructed with this same confidence level, sample size, etc.) that would contain the population proportion.
N is the size of the sample drawn from the population.
Distance from P to Upper Limit is the distance from the one-sided upper limit to the proportion.
Target Distance from P to Upper Limit is the value of the distance that is entered into the procedure.
Actual Distance from P to Upper Limit is the value of the distance that is obtained from the procedure.
Proportion (P) is the assumed sample proportion.
Lower Limit is the lower limit of the confidence interval.
Upper Limit is the upper limit of the confidence interval.
Distance from P to Limit if P = 0.5 is approximately the maximum distance from the one-sided upper limit to the sample proportion for a confidence interval with sample size N.

Summary Statements

A sample size of 169595 produces a one-sided 95% upper-limit confidence interval with a distance from the sample proportion to the upper limit that is equal to 0.002 when the sample proportion is 0.500.

Confidence Intervals for One Proportion - New

Chart Section

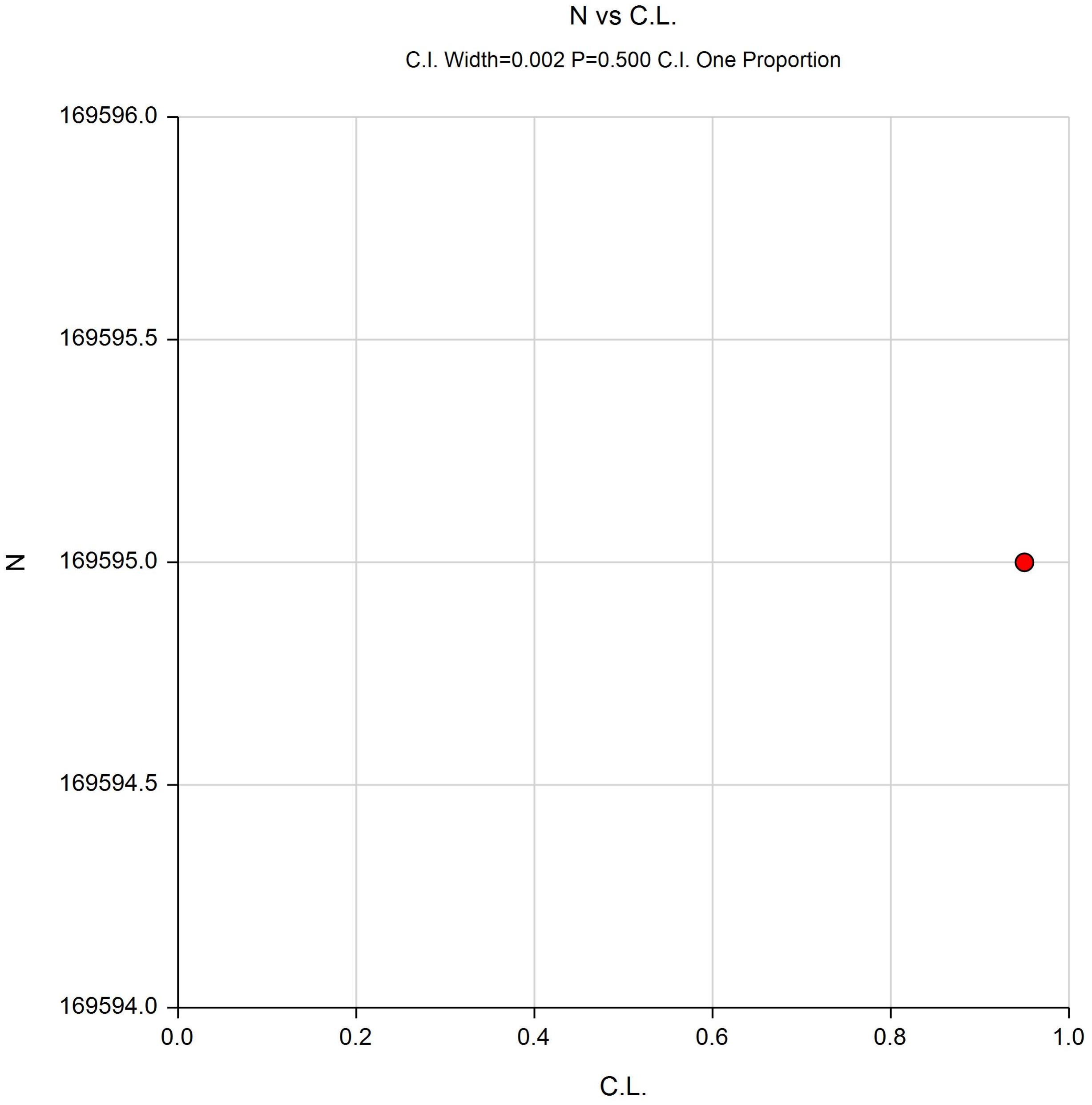

Supplement: Supplementary file 5 [file DataSheet_1.pdf]
